# Management of Coagulopathy in Patients with Decompensated Liver Cirrhosis

**DOI:** 10.4061/2011/695470

**Published:** 2011-11-17

**Authors:** Pooja D. Amarapurkar, Deepak N. Amarapurkar

**Affiliations:** ^1^Department of Gastroenterology, Bombay Hospital and Medical Research Centre, Mumbai 400 020, India; ^2^Ameya Co-Op Housing Society, New Prabhadevi Road, Prabhadevi, Mumbai 400 025, India

## Abstract

Patients with decompensated liver cirrhosis have significantly impaired synthetic function. Many proteins involved in the coagulation process are synthesized in the liver. Routinely performed tests of the coagulation are abnormal in patients with decompensated liver cirrhosis. This has led to the widespread belief that decompensated liver cirrhosis is prototype of acquired hemorrhagic coagulopathy. If prothrombin time is prolonged more than 3 seconds over control, invasive procedures like liver biopsy, splenoportogram, percutaneous cholangiography, or surgery were associated with increased risk of bleeding, and coagulopathy should be corrected with infusion of fresh frozen plasma. These practices were without any scientific evidence and were associated with significant hazards of fresh frozen plasma transfusion. Now, it is realized that coagulation is a complex process involving the interaction of procoagulation and anticoagulation factors and the fibrinolytic system. As there is reduction in both anti and procoagulant factors, global tests of coagulation are normal in patients with acute and chronic liver disease indicating that coagulopathy in liver disease is more of a myth than a reality. In the last few years, surgical techniques have substantially improved, and complex procedures like liver transplantation can be done without the use of blood or blood products. Patients with liver cirrhosis may also be at increased risk of thrombosis. In this paper, we will discuss coagulopathy, increased risk of thrombosis, and their management in decompensated liver cirrhosis.

## 1. Introduction

Traditionally decompensated liver cirrhosis has been considered as a prototype of hemorrhagic coagulopathy. Routinely performed coagulation profile is abnormal in the majority of these patients [1]. If prothrombin time is prolonged more than 3 seconds over control, invasive procedures like liver biopsy, splenoportogram, percutaneous cholangiography, or surgery were associated with increased risk of bleeding [2]. For years, it was evident that haemostasis tests performed in peripheral blood correlated poorly with the actual duration of bleeding and the amount of blood loss measured directly at laparoscopy from biopsy puncture. Abnormal bleeding after liver biopsy is a random event which cannot be predicted by currently used coagulation tests. Abnormal coagulation tests also did not correlate with development of soft tissue hematomas, variceal bleeding, and other bleeding episodes in cirrhotic patients [3–6]. 

In the recent years, surgical techniques have improved remarkably, and even liver transplantation can be performed without using blood or blood products [7]. It has been realized that thrombin plug formation is a dynamic process, and coagulation tests suggest that prothrombin time (PT) and activated partial thromboplastin time (APTT) explore only early phase of thrombin formation [8]. Thrombin formation is globally measured by using a thrombin generation assay modified by addition of thrombomodulin, and hence it is sensitive not only to the low plasma level of coagulation factors but also to the reduced levels of naturally occurring coagulation inhibitors in patients with liver disease. Patients with cirrhosis do form thrombin in amounts similar to healthy individuals [9]. Two single-theme conferences have been organized to address the issue of coagulation in patients with liver cirrhosis, and the reports of both meetings have been published [10, 11].

Both these reports suggest that coagulopathy in liver cirrhosis is a complex issue, and optimal strategies for prediction and prevention of bleeding episodes cannot be done by currently used coagulation tests and infusion of fresh frozen plasma. Strategies to treat bleeding complications in decompensated liver cirrhotic patients are not clear and require further clinical studies [10, 11]. The proper management of coagulopathy in patients with decompensated liver cirrhosis is highly debatable, and a major area of interest in the field of hepatology in this paper, the physiology of normal coagulation, the limitations of coagulation tests, and a reasonable approach to the management of coagulation disorders in patients with decompensated liver disease will be discussed.

## 2. Coagulation and Haemostatic Abnormalities in Context of Decompensated Liver Cirrhosis

Coagulation and heamostasis is a dynamic process with interplay between primary heamostasis, coagulation, and fibrinolysis. Majority of plasma clotting factors and proteins of the fibrinolytic and anticoagulants are synthesized in the liver, while cell surface factors (surface factor is a transmembrane protein that acts as a receptor and cofactor for FVII) responsible for heamostasis are not synthesized by liver. Normal coagulation system has been conceptualized as Y shape pathway with separate intrinsic and extrinsic component initiated factor XII or factor VIIa/tissue factors leading to a common pathway of factor Xa/factor Va. In patients with severe liver disease, heamostasis is affected due to diminished synthesis of factors II, V, VI, IX, X, XI, XIII, fibrinogen, protein C, protein S, Vitamin K deficiency due to malabsorption or malnutrition, dysfibrinogenemia, enhanced fibrinolysis, diffuse intravascular coagulation, thrombocytopenia, impaired clearance of activated clotting factors, plasminogen activators, and fibrinogen degradation products. Clinical consequences of this may lead to abnormal bleeding test, bleeding, and thrombosis. Coagulation in patients with decompensated liver cirrhosis can also be affected by other factors like infections, endogenous heparinoids, renal failure, and endothelial dysfunction [10–12]. Endogenous heparinoids have effect on coagulopathy in patients with cirrhosis. This has been demonstrated by thromboelastography with addition of heparinase 1 in patients who have recent variceal bleed or infection. Effect of endogenous heparinoids has also been seen after reperfusion of liver undergoing liver transplant patient [12]. 

Current concept of heamostasis is cell based. Primary heamostasis initiates from the adhesions of circulating platelets to the subendothelium at the site of injury through the mediation of the adhesive protein von Willebrand factor (VWF) and specific platelet receptors. Elevated levels of VWF seen in patients with cirrhosis are due to thrombocytopenia and decreased VWF protein, cleaving protease ADAMTS13 [13, 14]. After the adhesions, platelet aggregation due to fibrinogen or VWF with substances secreted by platelets themselves act as agonist. Activated platelets express their cell surface phosphatidylserine (P-serine) that promotes the conversion of factor II to thrombin by means of factor Xa, Va, and calcium. This is the initiating phase of a chain of events leading to thrombin generation and final conversion of fibrinogen to fibrin. Fibrin is stabilized factor XIII, and fibrinolysis is responsible for degradation of fibrin through a complex mechanism of pro- and antiactivators which regulates the generation of plasmin. Majority of the factors involved in heamostasis, coagulation, fibrinolysis, and anticoagulation are synthesized in the liver. In normal individuals, these systems are in a balance. In patients with cirrhosis, VWF plays a key role together with factor IX and negatively charged phospholipids of activated platelets to boost thrombin generation. Protein C activation by thrombin in complex with its endothelial receptor thrombomodulin acts as a powerful thrombin quenching protease by inhibiting the activated form of factor V and VIII. Patients with cirrhosis have increased levels of factor VIII and decreased levels of protein C and antithrombin. Elevation in factor VIII levels is due to decreased clearance from the circulation [8]. There are patients with isolated factor deficiencies like hemophilia and patients present with bleeding in contrast to patients with liver disease who have decreased levels of procoagulants and anticoagulants leading to a balance in the heamostasis without increasing the risk of bleeding, but this balance is precarious and may be tipped off either towards the bleeding or thrombosis by external factors like infection, renal failure, and so forth [11, 15]. 

Another important factor in coagulation in patients with decompensated liver cirrhosis is platelets. Patients with decompensated liver cirrhosis have thrombocytopenia and thrombocytopathy. This can be due to platelets sequestration, thrombopoietin deficiency, (myelosuppression due to hepatitis C, folate deficiency, and ethanol toxicity) autoantibodies, and low-grade disseminated intravascular coagulation (DIC) [16–18]. Standard diagnostic tests of platelet functions are of little use to predict the bleeding risk in patients with liver disease. Decrease in platelet function in cirrhosis is compensated by high plasma levels of VWF which compensates for platelet capacity to provide surface for thrombin generation. Platelet count beyond 50,000/mmq is adequate enough for a normal heamostasis. Prophylactic role of platelet transfusion is highly questionable [9].

### 2.1. Fibrinolysis and Liver Disease

Cirrhosis is considered to be a hyperfibrinolytic state. The fibrinolytic system consists of plasminogen which is converted to plasmin via intrinsic activation with factor XIIa, kallikrein, tissue plasminogen activator (tPA), and urokinase. All these factors are synthesized by the liver. Recent work has suggested that thrombin-activated fibrinolysis inhibitor (TAFI) is decreased in liver cirrhosis. Decrease in TAFI is counterbalanced by the concomitant decrease in profibrinolytic factors, and excessive fibrinolysis does not occur in patients with liver disease. Various tests are available for assessing fibrinolysis, and these include (1) clot lysis time, (2) euglobulin lysis time, (3) D-dimer assay, (4) fibrinogen degradation product assay, (5) tPA assay, and (6) thromboelastogram clot lysis index. Majority of these tests have high interindividual variability and low specificity. Except for thromboelastogram, no commercial test evaluates global fibrinolysis. Measurement of individual components of the fibrinolytic pathway is unlikely to help in assessing and managing bleeding risk of cirrhosis [19].

### 2.2. Clinical Tests for Coagulation in Liver Disease

A variety of coagulation of tests which include bleeding time, clotting time, prothrombin time, activated partial thromboplastin time, thrombin time, whole blood clot lysis, plasma fibrinogen, serum fibrinogen degradation product, plasma D-dimer, euglobulin lysis time, factor assays for F XIII, protein C, protein S, and antithrombin III. The literature evidence suggests that conventional coagulation tests are of little value in predicting bleeding risk in patients with cirrhosis and are of limited use in guiding decisions of the appropriate management of bleeding events in cirrhosis. For judging the risk of bleeding, we need tests for global evaluation of coagulation-like thrombin generation time, thromboelastography, sonorheometry, and national normalized ratio calibrated for cirrhosis (INRliver). Thrombin generation test is a global test in which coagulation cascade is activated with small amounts of tissue factor as a trigger and phospholipids acting as a platelets substitute. Thrombin generation measured in the presence of thrombomodulin and platelet-rich plasma is similar for patients with chronic liver disease and healthy subjects. The critical platelet count is 60,000/cumm [9, 20]. Thromboelastography measures clot formation, clot strength, and clot dissolution but does not measure vascular tone. It accesses global heamostasis. The modern thromboelastography which combines new computer technology with new materials and equipment is popular during surgical interventions like liver transplantation [21]. The INR liver is prothrombin time calibrated using plasma from patients with cirrhosis instead of vitamin K antagonists and may resolve variability of INR in these patients [22]. These tests have not been prospectively evaluated in patients with liver disease. Prothrombin time has been used traditionally in assessment of severity of liver disease in child pugh score or as a INR in MELD score. Prothrombin time expressed as an INR is highly variable and has never been standardized in patients with liver disease [20, 21]. Current controversies in patients with liver cirrhosis are which humoral or hematological test can predict risk of bleeding versus risk of thrombosis and which prophylactic intervention can be used effectively from both bleeding and thrombotic perspective. Recently, Tripodi et al. described a simple laboratory method which focuses on function of protein C deficiency which could promote clotting in patients of cirrhosis. This test is standardizable laboratory test which may determine the relative risk of clotting versus bleeding in patients with cirrhosis [23]. 

## 3. Management of Coagulopathy in Decompensated Liver Cirrhosis

Vitamin K deficiency is seen in decompensated liver cirrhosis secondary to various complex mechanisms which include bile salt deficiency, bile salt secretory failure, and use of broad spectrum antibiotics. 10 mg of vitamin K injections for three days is adequate enough to correct the vitamin K deficiency and should be given to patients with decompensated liver cirrhosis. Oral vitamin K has no role [1]. Prophylactic correction of prothrombin time using fresh frozen plasma is not recommended [22]. Prothrombin times more than 4 seconds over control are unlikely to get corrected with fresh frozen plasma. Fresh frozen plasma has unpredictable response in patients with decompensated liver cirrhosis and is associated with significant side effects like volume overload, exacerbation of portal hypertension, risk of infections, and risk of transfusion-related acute liver injury [7].

## 4. Management of Bleeding Episodes

Patients with bleeding should be investigated for superimposed causes of coagulopathy like infections, renal failure, and so forth. Superimposed insults should be corrected aggressively. Other therapeutic options are as shown in Table 1. Use of vitamin K has already been discussed. Platelet transfusion may be considered if platelet count is less than 50,000/mm^3^. Target platelet count to be achieved is more than 70,000/mm^3^. Fresh frozen plasma contains all coagulation factors, inhibitors of coagulation, and fibrinolytic factors. Fresh frozen plasma should be solvent detergent-treated plasma or donor-retested plasma. Therapeutic improvement is transient and may be associated with adverse reactions as mentioned above. Hypofibrinogenemia (fibrinogen <10 mg/dL) should be treated with cryoprecipitate till normal fibrinogen levels are reached. Other agents used in the treatment of fibrinolysis in patients with decompensated liver cirrhosis are aprotinin, transexaminc acid, and epsilon amino caproic acid. These agents play a major role in treating local bleeding but also carry a risk of thrombotic complications. Their use has not been well studied in clinical trials. Desmopressin (DDAVP) is an analogue of anti diuretic hormone vasopressin. DDAVP releases vWf and factor VIII. It shortens the bleeding time, and peak response is achieved in 30–60 minutes after intravenous administration. Unfortunately, no benefit of DDAVP administration is seen in patients with variceal bleeding and liver surgery. Recombinant activated factor VIIa is shown to improve prothrombin time and clot formation without enhanced fibrinolysis. The effect is immediate but transient. Repeated dosing is required and is extremely expensive. Recombinant factor VIIa though clearly corrects in vitro coagulation abnormalities was not shown to be effective in patients with variceal bleeding. Some advantage was shown in child C cirrhosis. The most efficient use of this product is in intracranial pressure monitor placement. It may have efficient role in controlling active variceal bleeding when there is no clear endoscopic view. Caveats with use of recombinant factor Va are thrombotic complications, high cost of the therapy, and limited outcome data. Control of bleeding can be achieved with topical haemostatic agents like fibrin glue, cyanoacrylates, thrombin, and suture supports. Surgical and anesthesiological methods used to reduce the blood loss during liver surgery in patients with cirrhosis are vascular clamping techniques, dissection devices like ultrasonic dissection, hydro jet dissection, thermal devises like argon plasma coagulator and radio frequency ablator, and topical haemostatic agents. Maintaining low central venous pressure and reducing the portal pressure may also be of help in controlling the bleeding during surgery [7, 8, 11].

Deep vein thrombosis, pulmonary embolism, and acute portal vein thrombosis can be treated with anticoagulants with special care [10]. Anticoagulation may prove to be safe and effective in patients with cirrhosis. Currently, anticoagulation has been used in patients with portal vein thrombosis and if thrombosis has extended to superior mesenteric vein. These patients are not suitable for liver transplantation. A recent randomized control trial has shown that low-molecular-weight heparin can prevent portal vein thrombosis and cirrhosis [24, 25].

In summary, heamostasis in patients with decompensated liver disease is a complex issue with counteracting forces which are in dynamic equilibrium and are affected by extraneous factors like infection and renal function. Currently, available tests for coagulation have a poor predictability for bleeding or thrombosis in patients with cirrhosis. New tests like TEG, thrombin tests, and so forth may give a better picture but need prospective studies. Role of specific intervention like platelet transfusion, antifibrinolytics and recombinant factors, anticoagulant need to be defined clearly.

## Figures and Tables

**Table 1:** Therapeutic options in coagulopathy in decompensated liver cirrhosis.

Agent	Utility in specific situations	Comment
Red blood cell transfusion	Bleeding patients	Transfusion should be minimum, not allowing Hb to exceed 8 to 9 mg%
Vitamin K	Every patient	May not be useful if patient has no deficiency
Fresh frozen plasma	Questionable in bleeding patients	May be used in bleeding patients when volume expansion is not a concern
Platelets	Count less than 50.000	Limited data
Cryoprecipitate	In bleeding patients	Limited data
Prothrombin complex concentrate	In bleeding patients	Limited data
Desmopressin	In bleeding patients	Efficacy unproved
Aprotinin, transexamine acid, and epsilon amino caprioric acid	Patients with hypofibrinogenemia Fibrinogen less than 100/dL	Can induce thrombosis
Recombinant factor VII	In placing ICP devices, bleeding after surgery, massive variceal bleed	Can induce thrombosis
Topical agents—cyanoacrylates, fibrin glue, and thrombin	Topical heamostasis and localized bleeding	Extremely expensive and limited data
Reduction in the portal pressure, maintaining low CVP by volume contraction (phlebotomy/diuresis)		
Surgical techniques—vascular clamping, ultrasonic/hydrojet dissectors, and thermal techniques (aarton plasma coagulator, radio frequency ablators)		
